# Crystal structure of (*E*)-*N*′-benzyl­idene-2-meth­oxy­benzohydrazide

**DOI:** 10.1107/S1600536814019011

**Published:** 2014-08-30

**Authors:** Muhammad Taha, M. Syukri Baharudin, Hamizah Mohd Zaki, Bohari M. Yamin, Humera Naz

**Affiliations:** a Atta-ur-Rahman Institute for Natural Product Discovery, Universiti Teknologi MARA (UiTM), Puncak Alam Campus, 42300 Bandar Puncak Alam, Selangor D.E., Malaysia; b Faculty of Applied Science, Universiti Teknologi MARA (UiTM), 40450 Shah Alam, Selangor D.E., Malaysia; c School of Chemical Sciences and Food Technology, Faculty of Science and Technology, Universiti Kabangsaan Malaysia, 43600 Bangi, Selangor, Malaysia; d Faculty of Pharmacy, Universiti Tecknologi MARA, Puncak Alam, 42300 Selangor, Malaysia

**Keywords:** crystal structure, benzohydrazide, Schiff base, hydrogen bonding

## Abstract

In the title benzoyl­hydrazide derivative, C_15_H_14_N_2_O_2_, the dihedral angle between the planes of the two phenyl rings is 12.56 (9)°. The azomethine double bond adopts an *E* configuration stabilized by an N—H⋯O hydrogen bond. In the crystal, the components are linked by C—H⋯O inter­actions to form chains along the *b* axis.

## Related literature   

For applications and biological activities of Schiff bases, see: Taha *et al.* (2013 ▶, 2014 ▶); Musharraf *et al.* (2012 ▶); Kaymakcioglu *et al.* (2006 ▶); Kucukguzel *et al.* (2003 ▶, 2004 ▶); Melnyk *et al.* (2006 ▶); Pandeya *et al.* (1999 ▶); Tarafder *et al.* (2002 ▶); Terzioglu & Gursoy (2003 ▶); Todeschini *et al.* (1998 ▶). For the crystal structures of related compounds, see: Taha *et al.* (2013 ▶).
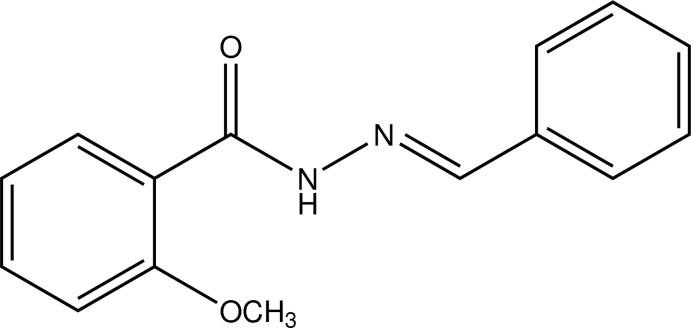


## Experimental   

### Crystal data  

C_15_H_14_N_2_O_2_
*M* *_r_* = 254.28
Orthorhombic, 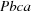

*a* = 13.3135 (9) Å
*b* = 9.9581 (6) Å
*c* = 20.0278 (14) Å
*V* = 2655.2 (3) Å^3^
*Z* = 8
Mo *K*α radiation
μ = 0.09 mm^−1^
*T* = 296 K
0.50 × 0.48 × 0.31 mm

### Data collection  

Bruker SMART APEX CCD area-detector diffractometer
Absorption correction: multi-scan (*SADABS*; Bruker, 2000 ▶) *T*_min_ = 0.94, *T*_max_ = 0.97
38941 measured reflections
2462 independent reflections
1819 reflections with *I* > 2σ(*I*)
*R*_int_ = 0.049

### Refinement  

*R*[*F* ^2^ > 2σ(*F* ^2^)] = 0.038
*wR*(*F* ^2^) = 0.103
*S* = 1.08
2462 reflections
174 parameters
H-atom parameters constrained
Δρ_max_ = 0.13 e Å^−3^
Δρ_min_ = −0.14 e Å^−3^

### 

Data collection: *SMART* (Bruker, 2000 ▶); cell refinement: *SAINT* (Bruker, 2000 ▶); data reduction: *SAINT*; program(s) used to solve structure: *SHELXTL* (Sheldrick, 2008 ▶); program(s) used to refine structure: *SHELXTL*; molecular graphics: *SHELXTL*; software used to prepare material for publication: *SHELXTL*, *PARST* (Nardelli, 1995 ▶) and *PLATON* (Spek, 2009 ▶).

## Supplementary Material

Crystal structure: contains datablock(s) global, I. DOI: 10.1107/S1600536814019011/bg2535sup1.cif

Structure factors: contains datablock(s) I. DOI: 10.1107/S1600536814019011/bg2535Isup2.hkl

Supporting information file. DOI: 10.1107/S1600536814019011/bg2535Isup3.cml

**Fig. 1.**
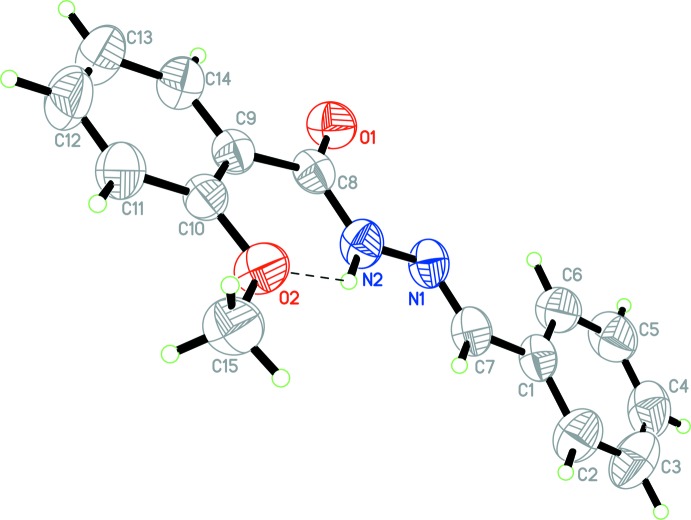
The molecular structure of (I) with displacement ellipsoids drawn at 50% probability level.

. DOI: 10.1107/S1600536814019011/bg2535fig1.tif
The mol­ecular structure of (I) with displacement ellipsoids drawn at 50% probability level.

**Fig. 2.**
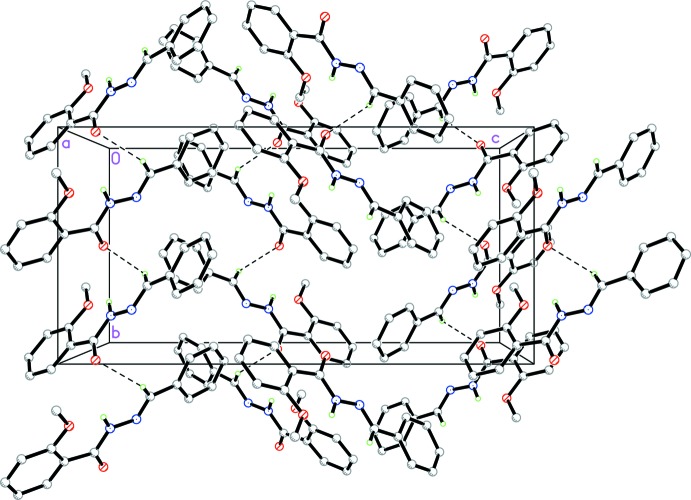
The crystal packing of the title compound I. Only hydrogen atoms involved in hydrogen bonding are shown.

. DOI: 10.1107/S1600536814019011/bg2535fig2.tif
The crystal packing of the title compound I. Only hydrogen atoms involved in hydrogen bonding are shown.

CCDC reference: 1020647

Additional supporting information:  crystallographic information; 3D view; checkCIF report

## Figures and Tables

**Table 1:** Hydrogen-bond geometry (Å, °)

*D*—H⋯*A*	*D*—H	H⋯*A*	*D*⋯*A*	*D*—H⋯*A*
N2—H2*A*⋯O2	0.86	1.96	2.6278 (17)	134
C7—H7*A*⋯O1^i^	0.93	2.44	3.1690 (19)	135

Symmetry code: (i) 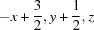.

## supplementary crystallographic information

### S1. Comment

Applications of benzoylhydrazones are reported in medicinal and analytical chemistry (Tarafder *et al.*, 2002). Benzoylhydrazones having heterocyclic rings have been reported to have antiglycation (Taha *et al.*, 2014), anticonvulsant (Kucukguzel *et al.*,2004), antiproliferative (Kucukguzel *et al.*, 2003), antifungal and anti-HIV activities (Pandeya *et al.*, 1999). Several benzoylhydrazones have also shown interesting bioactivities including antinflammatory (Todeschini *et al.*, 1998), antibacterial (Kaymakcioglu *et al.*, 2006), antimalarial (Melnyk *et al.*, 2006) and anticancer (Terzioglu & Gursoy, 2003). Recently they have been suggested as an alternative in UV-laser desorption ionization (LDI) matrices for peptides analysis (Musharraf *et al.*, 2012). The structure of the title compound (Fig. 1) is similar to (*E*)-2-methoxy-*N'*-(2,4,6-trihydroxybenzylidene) benzohydrazide (Taha *et al.*, 2013), the difference residing in that the trihydroxyphenyl ring has been replaced by a non-substitutedphenyl ring (C1–C6). The bond lengths and angle were found to be similar to the structurally related benzohydrazide derivatives reported in Taha *et al.*, 2013. The *E* configuration around the azomethine double bond is stabilized by a N2—H2A···O2 intramolecular interaction. The crystal structure is in turn stabilized by the intermolecular C7—H7A···O1 interaction to form chains along the *b* axis (Table 2 and Fig. 2).

### S2. Experimental

The title compound was synthesized by refluxing a mixture of 2 mmol of 2-methoxybenzohydrazide (0.332 g), 2 mmol benzaldehyde (0.212 g) and catalytic amount of acetic acid in methanol (20 ml) for 3 h. The progress of the reaction was monitored by TLC. After completion of the reaction, the solvent was evaporated by vacuum to afford crude product which was further recrystallized in methanol to afford needle like pure product in 88% yield (0.447 g). All the chemicals were purchased from sigma Aldrich Germany.

### S3. Refinement

H atoms were positioned geometrically with C—H = 0.93/0.96 Å, N-H = 0.86 Å respectively, and constrained to ride on their parent atoms with *U*_iso_(H)= 1.2/1.5 *U*_eq_(CH).

### Crystal data

**Table:**

C_15_H_14_N_2_O_2_	*D*_x_ = 1.272 Mg m^−^^3^
*M**_r_* = 254.28	Melting point = 449–451 K
Orthorhombic, *P**b**c**a*	Mo *K*α radiation, λ = 0.71073 Å
*a* = 13.3135 (9) Å	Cell parameters from 9899 reflections
*b* = 9.9581 (6) Å	θ = 3.0–25.5°
*c* = 20.0278 (14) Å	µ = 0.09 mm^−^^1^
*V* = 2655.2 (3) Å^3^	*T* = 296 K
*Z* = 8	Block, colourless
*F*(000) = 1072	0.50 × 0.48 × 0.31 mm

### Data collection

**Table:**

Bruker SMART APEX CCD area-detector diffractometer	2462 independent reflections
Radiation source: fine-focus sealed tube	1819 reflections with *I* > 2σ(*I*)
Graphite monochromator	*R*_int_ = 0.049
Detector resolution: 83.66 pixels mm^-1^	θ_max_ = 25.5°, θ_min_ = 3.0°
ω scan	*h* = −16→16
Absorption correction: multi-scan (*SADABS*; Bruker, 2000)	*k* = −11→12
*T*_min_ = 0.94, *T*_max_ = 0.97	*l* = −24→24
38941 measured reflections	

### Refinement

**Table:**

Refinement on *F*^2^	Primary atom site location: structure-invariant direct methods
Least-squares matrix: full	Secondary atom site location: difference Fourier map
*R*[*F*^2^ > 2σ(*F*^2^)] = 0.038	Hydrogen site location: inferred from neighbouring sites
*wR*(*F*^2^) = 0.103	H-atom parameters constrained
*S* = 1.08	*w* = 1/[σ^2^(*F*_o_^2^) + (0.0361*P*)^2^ + 0.849*P*] where *P* = (*F*_o_^2^ + 2*F*_c_^2^)/3
2462 reflections	(Δ/σ)_max_ < 0.001
174 parameters	Δρ_max_ = 0.13 e Å^−^^3^
0 restraints	Δρ_min_ = −0.14 e Å^−^^3^

### Special details

**Table:**

Geometry. All e.s.d.'s (except the e.s.d. in the dihedral angle between two l.s. planes) are estimated using the full covariance matrix. The cell e.s.d.'s are taken into account individually in the estimation of e.s.d.'s in distances, angles and torsion angles; correlations between e.s.d.'s in cell parameters are only used when they are defined by crystal symmetry. An approximate (isotropic) treatment of cell e.s.d.'s is used for estimating e.s.d.'s involving l.s. planes.
Refinement. Refinement of *F*^2^ against ALL reflections. The weighted *R*-factor *wR* and goodness of fit *S* are based on *F*^2^, conventional *R*-factors *R* are based on *F*, with *F* set to zero for negative *F*^2^. The threshold expression of *F*^2^ > σ(*F*^2^) is used only for calculating *R*-factors(gt) *etc*. and is not relevant to the choice of reflections for refinement. *R*-factors based on *F*^2^ are statistically about twice as large as those based on *F*, and *R*- factors based on ALL data will be even larger.

### Fractional atomic coordinates and isotropic or equivalent isotropic displacement parameters (Å^2^)

**Table:**

	*x*	*y*	*z*	*U*_iso_*/*U*_eq_	
O1	0.67310 (8)	−0.00143 (12)	0.55943 (6)	0.0569 (3)	
O2	0.92308 (8)	0.22579 (11)	0.51387 (6)	0.0575 (3)	
N1	0.69422 (9)	0.21778 (14)	0.63568 (6)	0.0481 (4)	
N2	0.75847 (10)	0.18784 (14)	0.58377 (6)	0.0486 (3)	
H2A	0.8086	0.2394	0.5750	0.058*	
C1	0.65053 (12)	0.36836 (17)	0.72316 (7)	0.0466 (4)	
C2	0.66885 (14)	0.4903 (2)	0.75366 (10)	0.0661 (5)	
H2B	0.7240	0.5414	0.7408	0.079*	
C3	0.60545 (17)	0.5369 (2)	0.80336 (11)	0.0797 (6)	
H3A	0.6183	0.6190	0.8238	0.096*	
C4	0.52403 (16)	0.4624 (2)	0.82254 (10)	0.0733 (6)	
H4A	0.4806	0.4946	0.8552	0.088*	
C5	0.50683 (15)	0.3407 (2)	0.79347 (10)	0.0701 (6)	
H5A	0.4523	0.2891	0.8071	0.084*	
C6	0.56950 (13)	0.29365 (18)	0.74411 (9)	0.0585 (5)	
H6A	0.5570	0.2105	0.7247	0.070*	
C9	0.80953 (11)	0.05112 (16)	0.48857 (7)	0.0434 (4)	
C14	0.78281 (13)	−0.05488 (19)	0.44740 (9)	0.0587 (5)	
H14A	0.7254	−0.1042	0.4573	0.070*	
C13	0.83879 (16)	−0.0892 (2)	0.39229 (10)	0.0727 (6)	
H13A	0.8196	−0.1609	0.3654	0.087*	
C12	0.92326 (16)	−0.0164 (2)	0.37740 (10)	0.0730 (6)	
H12A	0.9613	−0.0389	0.3401	0.088*	
C11	0.95232 (14)	0.08876 (19)	0.41671 (9)	0.0605 (5)	
H11A	1.0097	0.1374	0.4058	0.073*	
C10	0.89683 (11)	0.12330 (16)	0.47268 (8)	0.0452 (4)	
C15	1.00920 (14)	0.30448 (19)	0.49805 (10)	0.0662 (5)	
H15A	1.0165	0.3749	0.5304	0.099*	
H15B	1.0012	0.3431	0.4545	0.099*	
H15C	1.0679	0.2485	0.4986	0.099*	
C7	0.71491 (11)	0.32331 (17)	0.66852 (7)	0.0472 (4)	
H7A	0.7719	0.3726	0.6576	0.057*	
C8	0.74169 (11)	0.07632 (16)	0.54683 (7)	0.0431 (4)	

### Atomic displacement parameters (Å^2^)

**Table:**

	*U*^11^	*U*^22^	*U*^33^	*U*^12^	*U*^13^	*U*^23^
O1	0.0488 (7)	0.0567 (7)	0.0652 (8)	−0.0047 (6)	0.0131 (6)	0.0040 (6)
O2	0.0509 (7)	0.0553 (7)	0.0664 (8)	−0.0085 (5)	0.0210 (6)	−0.0075 (6)
N1	0.0414 (7)	0.0615 (9)	0.0413 (7)	0.0032 (6)	0.0088 (6)	0.0011 (7)
N2	0.0402 (7)	0.0594 (9)	0.0461 (7)	−0.0032 (6)	0.0126 (6)	−0.0014 (7)
C1	0.0436 (9)	0.0579 (10)	0.0383 (8)	0.0066 (8)	−0.0003 (7)	0.0039 (7)
C2	0.0573 (11)	0.0763 (13)	0.0649 (11)	−0.0044 (10)	0.0020 (9)	−0.0128 (10)
C3	0.0799 (14)	0.0884 (15)	0.0708 (13)	0.0113 (12)	0.0001 (12)	−0.0298 (12)
C4	0.0756 (14)	0.0940 (16)	0.0504 (11)	0.0258 (12)	0.0128 (10)	−0.0019 (11)
C5	0.0690 (13)	0.0747 (14)	0.0665 (12)	0.0094 (10)	0.0263 (10)	0.0095 (11)
C6	0.0602 (11)	0.0581 (10)	0.0574 (10)	0.0037 (8)	0.0167 (9)	0.0015 (9)
C9	0.0390 (8)	0.0476 (9)	0.0435 (9)	0.0065 (7)	0.0018 (7)	0.0047 (7)
C14	0.0561 (11)	0.0653 (11)	0.0547 (10)	−0.0044 (9)	0.0022 (8)	−0.0046 (9)
C13	0.0794 (14)	0.0804 (14)	0.0584 (12)	−0.0032 (12)	0.0064 (10)	−0.0198 (10)
C12	0.0764 (13)	0.0871 (15)	0.0556 (11)	0.0023 (12)	0.0211 (10)	−0.0119 (11)
C11	0.0577 (11)	0.0671 (12)	0.0569 (11)	0.0002 (9)	0.0193 (9)	0.0003 (9)
C10	0.0444 (9)	0.0465 (9)	0.0446 (9)	0.0079 (7)	0.0063 (7)	0.0031 (7)
C15	0.0567 (11)	0.0658 (12)	0.0762 (12)	−0.0137 (9)	0.0166 (9)	−0.0003 (10)
C7	0.0382 (8)	0.0613 (11)	0.0420 (8)	0.0012 (8)	0.0029 (7)	0.0044 (8)
C8	0.0357 (8)	0.0489 (9)	0.0447 (8)	0.0063 (7)	0.0019 (7)	0.0078 (7)

### Geometric parameters (Å, º)

**Table:**

O1—C8	1.2236 (18)	C5—H5A	0.9300
O2—C10	1.3579 (19)	C6—H6A	0.9300
O2—C15	1.424 (2)	C9—C14	1.386 (2)
N1—C7	1.270 (2)	C9—C10	1.403 (2)
N1—N2	1.3790 (17)	C9—C8	1.497 (2)
N2—C8	1.353 (2)	C14—C13	1.375 (2)
N2—H2A	0.8600	C14—H14A	0.9300
C1—C6	1.376 (2)	C13—C12	1.371 (3)
C1—C2	1.381 (2)	C13—H13A	0.9300
C1—C7	1.461 (2)	C12—C11	1.366 (3)
C2—C3	1.385 (3)	C12—H12A	0.9300
C2—H2B	0.9300	C11—C10	1.386 (2)
C3—C4	1.368 (3)	C11—H11A	0.9300
C3—H3A	0.9300	C15—H15A	0.9600
C4—C5	1.364 (3)	C15—H15B	0.9600
C4—H4A	0.9300	C15—H15C	0.9600
C5—C6	1.376 (2)	C7—H7A	0.9300
			
C10—O2—C15	119.05 (13)	C13—C14—H14A	119.1
C7—N1—N2	115.77 (13)	C9—C14—H14A	119.1
C8—N2—N1	119.16 (13)	C12—C13—C14	119.22 (19)
C8—N2—H2A	120.4	C12—C13—H13A	120.4
N1—N2—H2A	120.4	C14—C13—H13A	120.4
C6—C1—C2	118.61 (16)	C11—C12—C13	120.82 (18)
C6—C1—C7	121.51 (16)	C11—C12—H12A	119.6
C2—C1—C7	119.86 (16)	C13—C12—H12A	119.6
C1—C2—C3	120.31 (19)	C12—C11—C10	120.36 (17)
C1—C2—H2B	119.8	C12—C11—H11A	119.8
C3—C2—H2B	119.8	C10—C11—H11A	119.8
C4—C3—C2	120.2 (2)	O2—C10—C11	122.73 (15)
C4—C3—H3A	119.9	O2—C10—C9	117.41 (13)
C2—C3—H3A	119.9	C11—C10—C9	119.86 (16)
C5—C4—C3	119.64 (18)	O2—C15—H15A	109.5
C5—C4—H4A	120.2	O2—C15—H15B	109.5
C3—C4—H4A	120.2	H15A—C15—H15B	109.5
C4—C5—C6	120.5 (2)	O2—C15—H15C	109.5
C4—C5—H5A	119.8	H15A—C15—H15C	109.5
C6—C5—H5A	119.8	H15B—C15—H15C	109.5
C5—C6—C1	120.70 (18)	N1—C7—C1	120.99 (15)
C5—C6—H6A	119.7	N1—C7—H7A	119.5
C1—C6—H6A	119.7	C1—C7—H7A	119.5
C14—C9—C10	117.90 (15)	O1—C8—N2	122.00 (14)
C14—C9—C8	115.89 (14)	O1—C8—C9	120.33 (15)
C10—C9—C8	126.21 (14)	N2—C8—C9	117.66 (14)
C13—C14—C9	121.84 (17)		
			
C7—N1—N2—C8	179.48 (14)	C12—C11—C10—O2	179.16 (17)
C6—C1—C2—C3	−1.2 (3)	C12—C11—C10—C9	−0.9 (3)
C7—C1—C2—C3	176.93 (17)	C14—C9—C10—O2	−179.12 (14)
C1—C2—C3—C4	−0.1 (3)	C8—C9—C10—O2	0.2 (2)
C2—C3—C4—C5	1.4 (3)	C14—C9—C10—C11	0.9 (2)
C3—C4—C5—C6	−1.4 (3)	C8—C9—C10—C11	−179.76 (15)
C4—C5—C6—C1	0.0 (3)	N2—N1—C7—C1	177.81 (13)
C2—C1—C6—C5	1.3 (3)	C6—C1—C7—N1	4.7 (2)
C7—C1—C6—C5	−176.83 (16)	C2—C1—C7—N1	−173.47 (16)
C10—C9—C14—C13	−0.4 (3)	N1—N2—C8—O1	−2.5 (2)
C8—C9—C14—C13	−179.76 (17)	N1—N2—C8—C9	176.67 (13)
C9—C14—C13—C12	−0.2 (3)	C14—C9—C8—O1	6.6 (2)
C14—C13—C12—C11	0.3 (3)	C10—C9—C8—O1	−172.75 (15)
C13—C12—C11—C10	0.3 (3)	C14—C9—C8—N2	−172.60 (14)
C15—O2—C10—C11	2.2 (2)	C10—C9—C8—N2	8.1 (2)
C15—O2—C10—C9	−177.76 (15)		

### Hydrogen-bond geometry (Å, º)

**Table:**

*D*—H···*A*	*D*—H	H···*A*	*D*···*A*	*D*—H···*A*
N2—H2*A*···O2	0.86	1.96	2.6278 (17)	134
C7—H7*A*···O1^i^	0.93	2.44	3.1690 (19)	135
C14—H14*A*···O1	0.93	2.39	2.730 (2)	101

Symmetry code: (i) −*x*+3/2, *y*+1/2, *z*.
