# Supplementary figures and images for: Crystal structure of (E)-N′-benzyl­idene-2-meth­oxy­benzohydrazide
Source: Acta Crystallogr Sect E Struct Rep Online. 2014 Aug 30;70(Pt 9):o1071–2. doi: 10.1107/S1600536814019011 (PMC4186185; doi:10.1107/S1600536814019011)

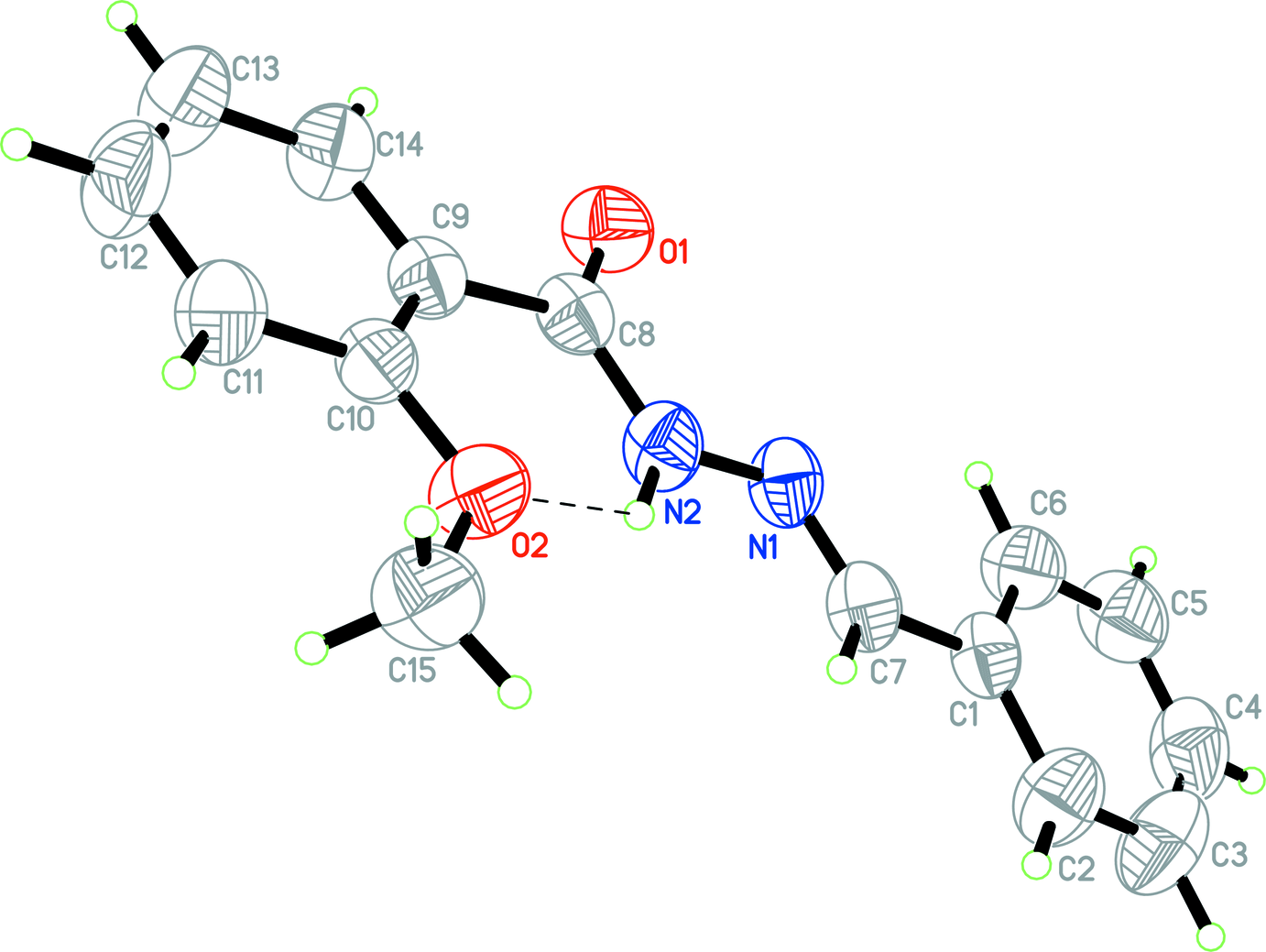

Supplement: Supplementary file 4 [file e-70-o1071-fig1.tif]

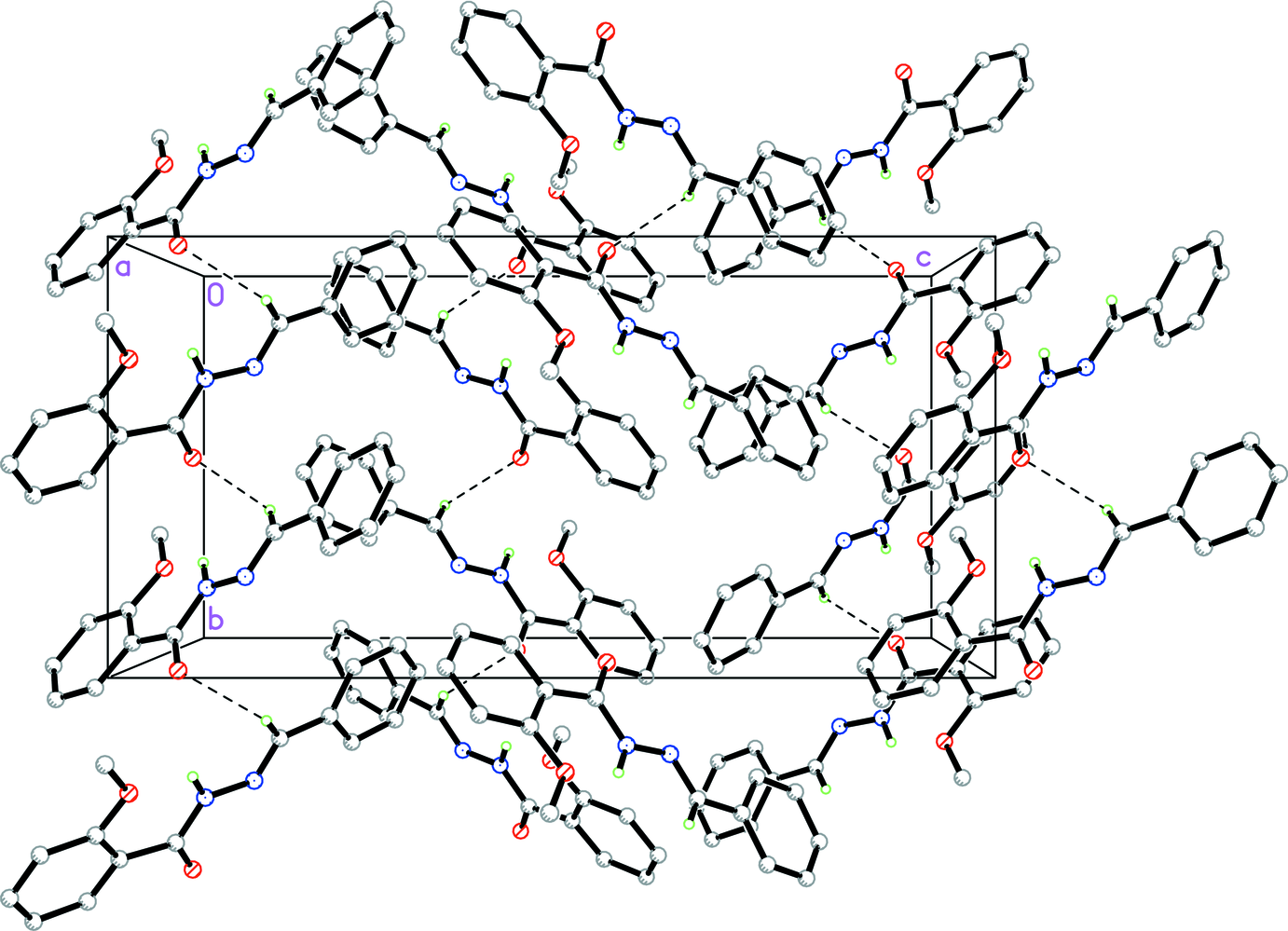

Supplement: Supplementary file 5 [file e-70-o1071-fig2.tif]
